# Supplementary material for: Metformin Attenuates Hyperglycaemia-Stimulated Pro-Fibrotic Gene Expression in Adventitial Fibroblasts via Inhibition of Discoidin Domain Receptor 2
Source: Int J Mol Sci. 2022 Dec 29;24(1):585. doi: 10.3390/ijms24010585 (PMC9820506; doi:10.3390/ijms24010585)
Supplement: Supplementary file 1 [file ijms-24-00585-s001.zip › ijms-2032866-supplementary.pptx]

## Slide 1
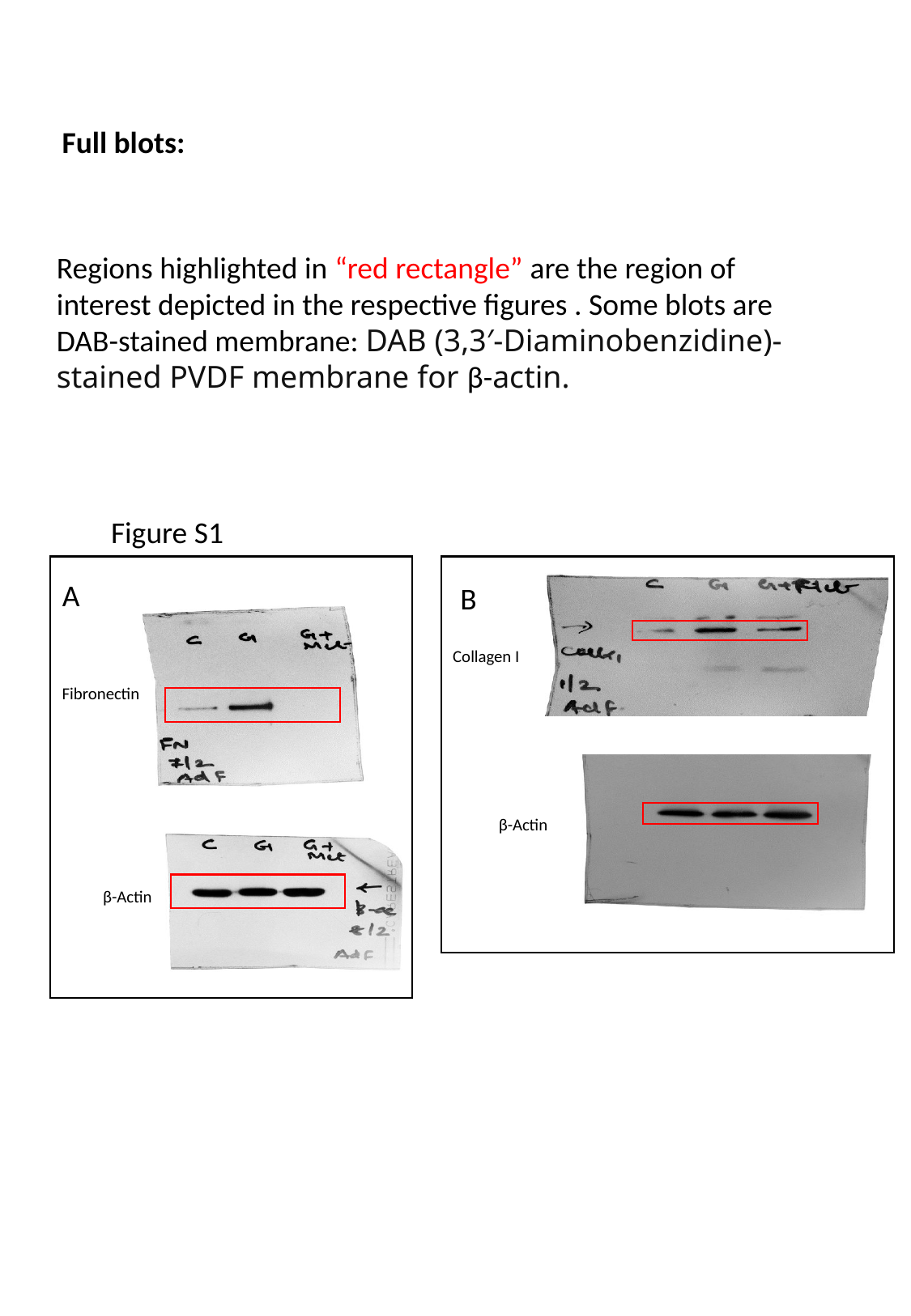

Full blots:
Regions highlighted in “red rectangle” are the region of interest depicted in the respective figures . Some blots are DAB-stained membrane: DAB (3,3′-Diaminobenzidine)-stained PVDF membrane for β-actin.
Figure S1
A
B
Collagen I
Fibronectin
β-Actin
β-Actin

## Slide 2
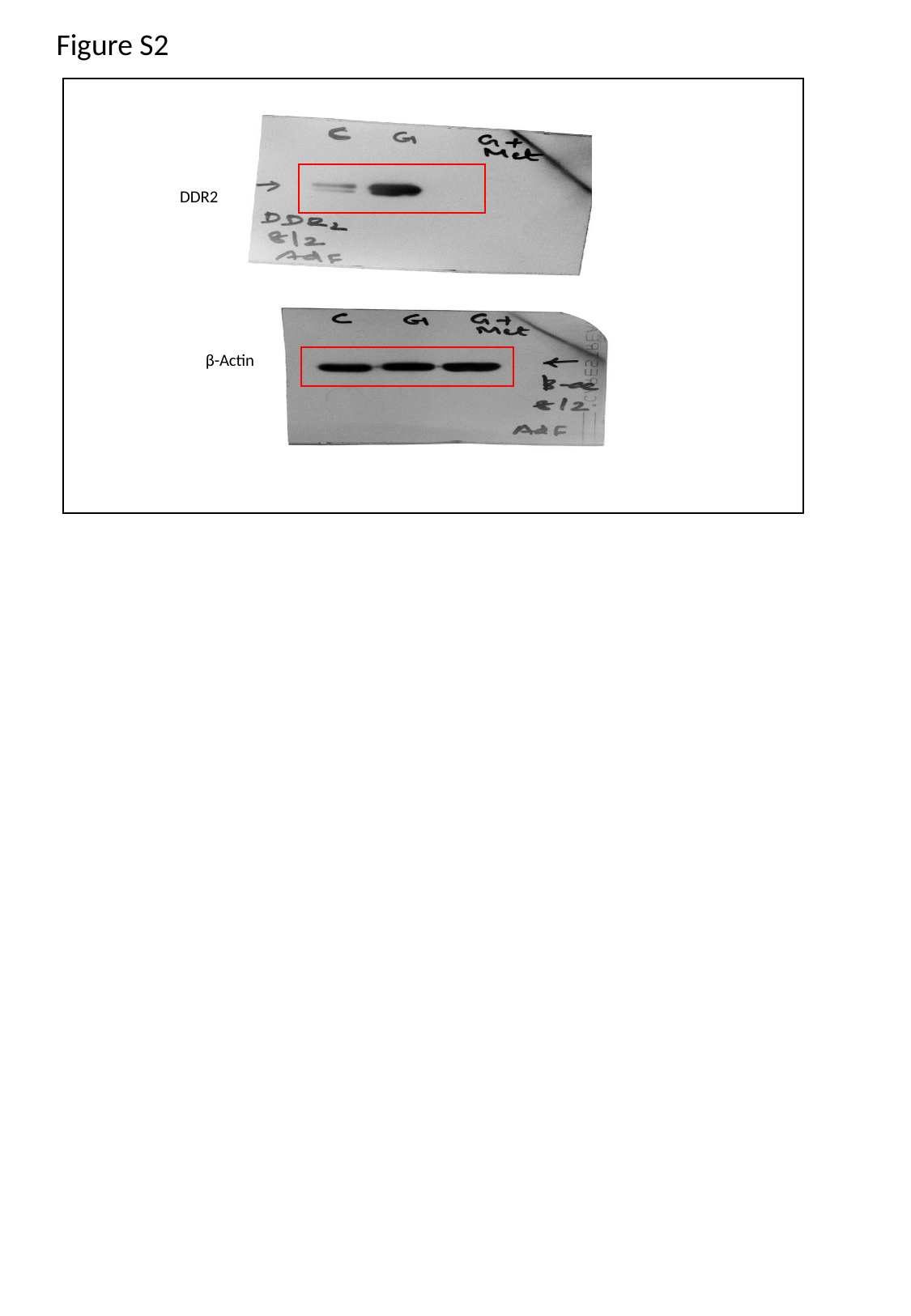

Figure S2
DDR2
β-Actin

## Slide 3
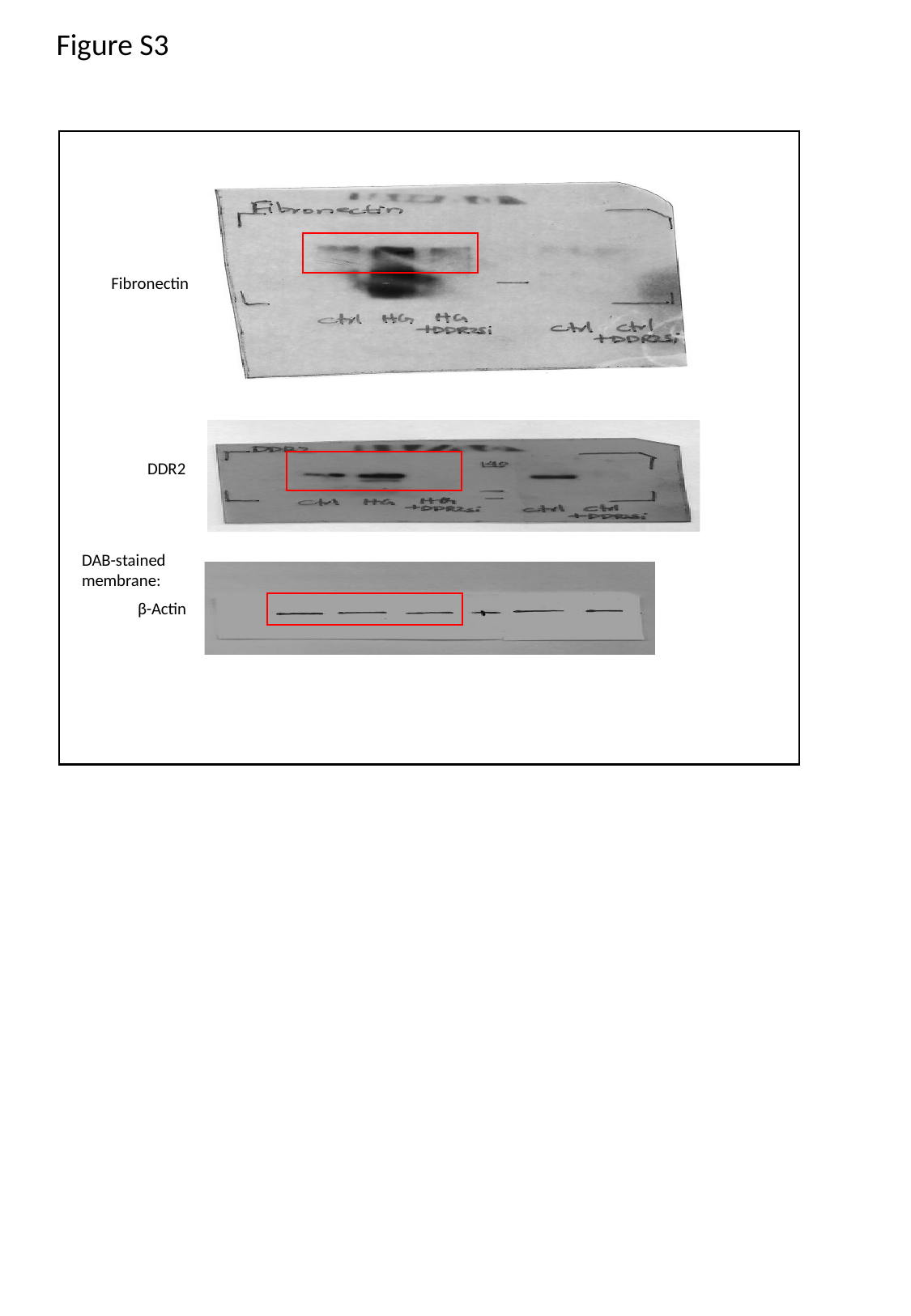

Figure S3
Fibronectin
DDR2
DAB-stained membrane:
β-Actin

## Slide 4
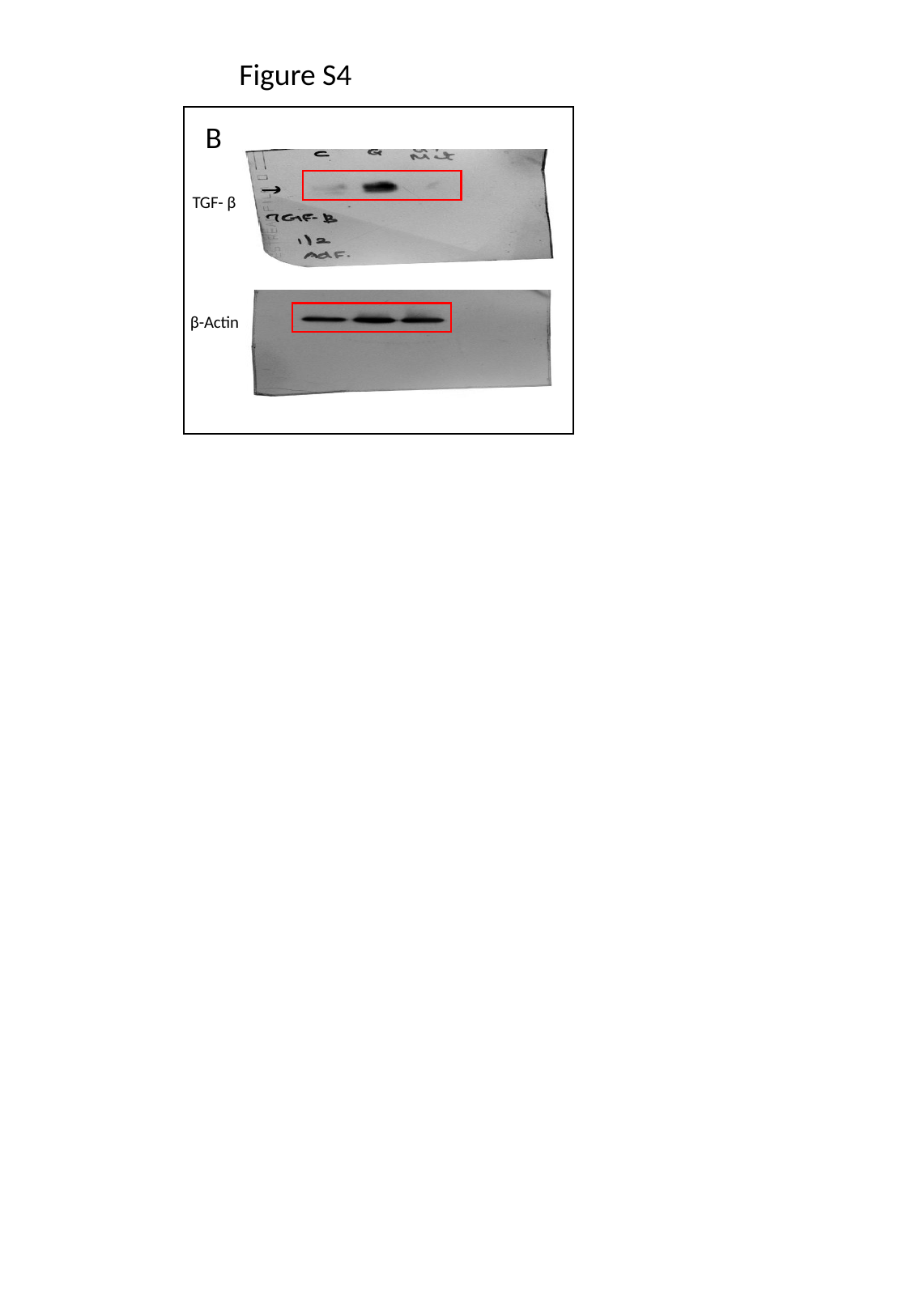

Figure S4
B
TGF- β
β-Actin

## Slide 5
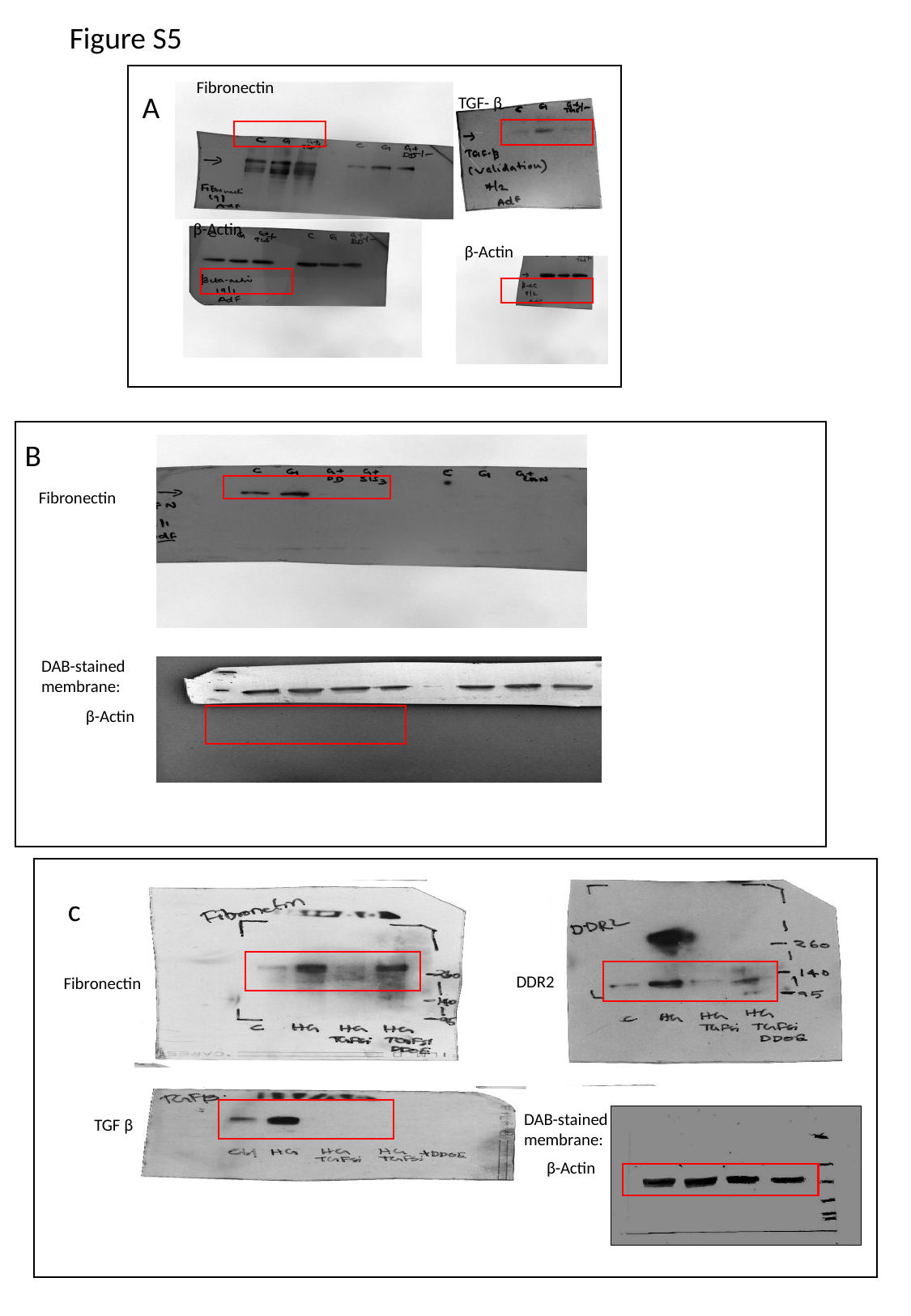

Figure S5
Fibronectin
TGF- β
β-Actin
β-Actin
A
B
Fibronectin
DAB-stained membrane:
β-Actin
c
DDR2
Fibronectin
DAB-stained membrane:
TGF β
β-Actin
